# Book and Pamphlet Notices

**Published:** 1860-11

**Authors:** 


### Editorial.

### BOOK AND PAMPHLET NOTICES.

On the Diseases, Injuries, and Malformations of the Rectum and Anus; with Remarks on Habitual Constipation. By T. I. Ashton, Surgeon to the Blenheim Dispensary, etc. From the Third and Enlarged English Edition. Philadelphia: Blanchard & Lea; 1860, pp. 292.

A good work on the rectum was a desideratum in medical literature, for it is true, as stated by Mr. Ashton, in his preface, that the diseases of this part "have been but superficially noticed by surgical writers generally, and but imperfectly understood by many of the profession."

The remarks on the best method of exploring the rectum, where more than a digital examination is required, are judicious. He advises the use of the coarse or rectum speculum, with an opening on one side, or that with two blades. Perhaps the following directions on the subject of enemata, may be found of service:

"Enemata, in most affections of the rectum, as well as in many other diseases, are productive of the greatest benefit, more effectually accomplishing the object of the physician in removing accumulated excretions than any other means, and saving the stomach and commencement of the intestinal canal from the irritation and nausea which aperient medicines induce. Whatever the form of the instrument, it is important that the jet should be elastic, and not, as usually supplied by instrument makers—made of ivory or metal, by which laceration or any other injury of the bowel is very readily inflicted. Pumps are objectionable for the reasons that patients are apt to throw up too large or too small a quantity of fluid, the necessity of a basin or other receptacle, and the inconvenience of employing both hands. From their simplicity and convenience, I recommend either a ten ounce India-rubber bottle with a stop-cock, or a cylindrical reservoir fitted with a piston: the jet is seven or eight inches in length, and being detached, affords the important advantages of great facility of introduction into the bowel: and by means of a plug, its connection with the instrument is most readily affected. When it is intended by enemata to unload the colon of accumulated fœcal matter impacted in its sacculi and distending that intestine, a long, elastic tube, known as "O'Beirne's tube," should be passed up the bowel, and the fluid injected by means of a well made double action pump. Before using the injecting apparatus it should be filled with fluid, otherwise the air contained will be forced into the patient's bowels, and cause much pain and annoyance; this precaution is highly necessary, for it is astonishing how much suffering will be induced if it is disregarded."

On the subject of irritation and itching of the anus, an affection often found so difficult of cure, the views of Mr. Ashton are worthy of note:

"In the treatment of this very troublesome and frequently obstinate disease, great patience and perseverence will often be requisite, both on the part of the patient and medical attendant. By the latter it must be borne in mind that the affection is rather a symptom of constitutional derangement than a disease *sui generis*, therefore the first endeavor must be to ascertain the cause producing it. In females, when the menstrual function has ceased, or is about to do so, it will be most important to keep the bowels free, to attend to the secretion of the liver, kidneys, and skin, and to direct exercise in the open air to be taken daily. If ascarides in the rectum give rise to the affection, they must be dislodged by such means as are recommended in treating of the subject under the head of foreign bodies in the rectum. If hemorrhoidal tumors or condylomata exist, they must be removed by excision, unless the hemorrhoids are internal, in which case the ligature or concentrated nitric acid must be employed. If the digestive and assimilative functions of the patient are feeble, and the constitution is otherwise delicate, a nutricious but plain diet will be necessary, conjoined with proper exercise, and the administration of alterative, tonic, and chalybeate medicines; but if the contrary be the case, and he has been accustomed to indulg in highly seasoned dishes, and to partake freely of wine aud spiritous liquors, he must be restricted to a vegetable diet, and the quantity of the stimuli considerably reduced, if not altogether disallowed. Various remedies have been recommended in this disease, and will be found more or less efficacious, according to the circumstances of the case; among them may be mentioned the decoction and infusion of cinchona with nitric or nitro-hydrychloric acid, and the various preparations of iron: the bowels must be acted on by the occasional use of purgatives. When an eruption exists on other parts of the body, five grains of the componnd pill of chloride of mercury should be taken at bed time, or the same quantity of mercury and chalk with hyoscyamus, coninm, or extract of poppy; and the compound decoction of sarsaparilla two or three times a day: when the gums become tender, the quantity of mercury must be reduced, or even left off for a short time, as ptyalism to any extent must be avoided. It will be advisable to continue the remedies for a few weeks after the disease has subsided, in order to guard against a relapse.

"The due attention to the functions of the skin has been insisted on, and much advantage as well as comfort will be derived from the use of the warm bath every second or third day.

"The local remedies that will be found useful are lotions containing acetate of lead with wine of opium, the bicyanide of mercury in bitter-almond mixture, lime water and calomel, or the bichloride of mercury, or a saturated solution of biborate of soda, ointments of lead, zinc, nitrate of mercury, &c.; but that which will frequently be found most serviceable, is the application to the part of a solution of nitrate of silver sufficiently diluted not to produce vesication, but only to excite a slight exfoliation of the skin."

Passing over the chapter on excresences, as being of less general interest, we come to that on contraction of the anus which sometimes exists without being suspected. Contraction may be congenital, may result from wounds and operations, particularly those occasioning loss of substance, from venereal disease, from inflammatory action, and it is often accompanied by fissure, either as cause or effect. This enumeration of the causes is sufficient in itself to put the surgeon on his guard against removing, unnecessarily, any portion of skin or mucous membrane in operations for fistula or piles.

"The treatment must be both medical and surgical. If inflammatory action be present, it must be subdued by topical blood-letting, hot fomentations, and cataplasms. The bowels must in all cases be kept loose by laxatives, as castor oil, confection of senna, &c.; great ease will be afforded by emollient enemata. The diet must be very moderate in quantity, and unstimulating in quality. The anus must be dilated by the introduction of bougies, and must be effected with much gentleness, for more pain will be experienced in this disease than in stricture of the rectum, in consequence of the greater sensibility of the integument than of the mucous membrane. When the instrument is used, the patient should rest on a couch or bed, with his knees drawn up. The better time for passing the bougie will be shortly before the usual period of the bowel's acting. Ablutions with soap and water, twice or thrice a day, will add to the patient's comfort, and lessen the local irritation. If much pain and nervous excitement be occasioned, anodynes may be required, which may be administered either by the mouth, or as suppositories, or enemata."

The chapter on hemorrhoids or piles is interesting, and we propose to make extracts sufficient to enable the reader to form an estimate of the author's views of pathology and treatment:

"*External Hemorrhoids.*—These tumors occur at the verge of the anus, and are covered by the thin integument of that region; but occasionally they will be observed to extend a short distance within the anal orifice, and will then be partly covered by the integument, and partly by the mucous membrane of the intestine. In form they are mostly globate, and have a broad extended base; they are of a livid color at first, but lose that as their active state subsides. They are tense and elastic to the touch, and exquisitely painful when inflamed, the anguish then being so great that the patient is unable to walk or take any exercise: in some cases even sitting is impossible. They consist of the integument and cellular tissue into which blood has been extravasated, as a result of the congested state of the hemorrhoidal vessels and determination of blood to them, produced by causes to be hereafter mentioned; generally, the blood is encysted in a central cavity, having a smooth glistening surface; in some cases there are several of these cavities filled with blood.

"After the acute stage attending the development of these tumors has subsided, the blood that has been effused into their interior becomes absorbed, and if they have not been distended to any great extent the skin contracts, and the parts resume their natural condition; but if the tumors have attained the size of a cherry, or larger, the elasticity of the integument will have been destroyed by over-distention, and upon absorption of their fluid contents pendulous flaps remain, prone to take on increased action, and form excrescences which may attain a considerable size, and occasion as much or more suffering than the primary disease."

"*Internal Hemorrhoids.*—The tumors constituting internal piles, consist of a morbid alteration in some portion of the mucous membrane of the rectum, and submucous areolar tissue, with an augmented and abnormal development of the capillary vessels. Like the external variety, they were formerly considered to be a dilatation of the veins. It appears somewhat surprising that this opinion should have been retained by many of the later writers; for when speaking of the character of the hemorrhage, they described it as florid and bright, and more nearly resembling arterial than venous blood, which it would not if it were poured out from veins, particularly when they are in a dilated and debilitated condition: in them the circulation must necessarily be slow, and consequently the blood would acquire a deeper color. But examinations on the living subject, and dissections on the dead, clearly demonstrate a different condition. A varicose state of the hemorrhoidal veins is not unfrequently met with; however, they form tumors very different in character, and in the symptoms they occasion, from those now under consideration.

"By dissection, internal hemorrhoidal tumors will be found to consist of both arteries and veins, the latter capacious, not in a diseased condition, but merely of abnormal development; the areolar tissue of the mucous membrane is hypertrophied, and if the tumors have existed long, and been subject to repeated inflammatory attacks, it will also be condensed. The surface of these tumors is frequently villous, presenting to the unassisted eye a granular appearance: they generally bleed freely if rudely touched, or accidentally scratched by the nail during an examination, the blood being of a bright red color. Dr. Bushe states that he has been able to rub off an exceedingly vascular and fragile and adventitious membrane from their surface, and is of opinion they may thus acquire an increase in magnitude. To the touch they have a spongy, elastic feel, and by some authors are considered to resemble erectile tissue in structure; had they compared them to those abnormal developments of the vascular system termed aneurism by anastomosis, the analogy would have been more correct.

"Internal hemorrhoids vary much in size and number, but the accessory phenomena attending them, such as pain, hemorrhage, &c., are not increased in proportion to either, and cases are met with in which a greater loss of blood occurs, or a greater amount of pain and suffering is induced from one or two small piles, than when there are several large ones.

"When one of these tumors is situated near the anus, though it may not have attained any great size, it is very liable to be prolapsed during defecation, particularly if the bowels are costive, giving rise to pain, spasm of the sphincter, and other distressing symptoms. Those that are situated higher in the bowel are not prolapsed so early in the disease; but, by repeated irritation and the dragging down they experience during the time the fæces are evacuated, they become elongated, and at length protrude externally: at first they return within the anus, by the action of the muscles of the part, but after a time the patient finds he is obliged to replace them with his fingers. In some cases this is done with facility, but others present where greater difficulty is experienced, owing either to the size of the tumors, or their being constricted by the sphincter muscle: under these circumstances the suffering is very great, and the individual is induced to postpone the calls of nature, or defer them till the night, finding it easier to return the tumors whilst he is in a horizontal position, in which he also experiences more speedy relief from pain. In many many cases, when the tumors are large and numerous, and have been subject to prolapse for a length of time, the sphincter and tissues of the anus lose their tone, are much relaxed, and the patient is subject to constant annoyance by their protrusion whenever he attempts to walk, or even by riding in a carriage: nor is the prolapsus in this stage confined to the tumor alone, for the bowel, having lost its support, the pouch of the rectum is easily dragged down by the morbid growths, and by the expulsive efforts at stool."

Hemorrhage is one of the most frequent of the accessory phenomena of internal piles, and at times the most prominent symptom, and, when excessive, is also the most alarming, from the serious effects thereby occasioned: it usually takes place during the evacuation of the contents of the bowel, occurring after the passage of the fœces, but sometimes preceding them. It is mostly of an active character, but may become passive by the vessels being debilitated, and the blood attenuated, as a consequence of the profuseness of the hemorrhagic discharge. The color of the blood evacuated is bright vermilion, and is exuded by the capillary vessels of the mucous membrane of the tumors or excrescences constituting the piles: this will be very evident on examination when they are prolapsed. In other cases the blood will be projected in fine streams, as if from minute vessels or dilated pores; but we are not able to detect these after the hemorrhage ceases."

"The sufferings and inconvenience to a patient affected with internal piles are often greatly increased by their protruding external to the anus. When the tumors are situated immediately within the rectum, they are subject to prolapsus in an earlier stage of the disease, owing to the eversion of the lower part of the mucous membrane, which occurs at the time of emptying the bowels, and to the fæces thrusting the tumors before them; when situated higher up in the intestine, they do not descend at so early a period, but, by the pressure and elongation they are subject to from the passage of the fæces, they at length protrude externally. At first the piles are retracted within the anus by muscular action alone after the bowels have been relieved; but in process of time this no longer occurs, and it becomes necessary to return them. Another source of distress from the prolapsus of piles, is their liability to strangulation, either by the spasmodic contraction of the sphincter, or by sanguineous engorgement: under these circumstances, the assistance of a surgeon will be required to effect the replacement of the extruded parts. If the patient delays seeking the necessary aid, mortification takes place, endangering his life should the constitution be impaired by any cause, or the vital powers be naturally feeble; if the contrary condition exists and the general health be good, the tumors will slough off, and a cure will thus be effected, but at the expense of much suffering."

"Any impediment offered to the return of the blood from the lower bowel will cause hemorrhoids. It will arise from two causes, the one being mechanical in its immediate effect, the other pathological, and depending on disease and alteration of structure in some of the internal organs. Those causes which act mechanically are the pregnant uterus, and other tumors developed in the pelvis or abdomen, which, by pressure on the large venous trunks, impede the ascent of the blood; tight lacing and cinctures also have the same effect. The pathological causes are congestion and structural diseases of the liver, pancreas, and spleen; diseases of the lungs, heart, and large bloodvessels, interfering with the free circulation of the blood.

"Hemorrhoids are frequently a concomitant of pregnancy, and in this state are of the accidental or occasional form, being induced by the gravid uterus pressing on the venous trunks, and by the general plethora which exists at this period.

"Constipation is one of the most frequent and common causes of hemorrhoids which we meet with: it tends to induce the disease in several way; thus, when the fæces are retained, they become indurated and inpacted, and produce irritation of the mucous membrane, and consequent afflux of blood to the rectum; by accumulation they distend the intestine, and, pressing on the viens, interfere more or less with the return of the blood. In this habit of body the hemorrhoidal vessels become greatly engorged during the act of defecation, from the violent efforts of the expulsatory muscles, and the congestion, arising during the temporary suspended respiration that always attends violent muscular action.

"Those persons whose habits of life are sedentary, are very generally the subject of piles, more especially if they ind[illegible]ge freely at table. By inactivity of body, the functions of the several emunctory organs are diminished, and, not the least important, that of the skin, which, when properly performed, frees the system of the effects of the effete tissues, which, if retained, have a most pernicious effect on the animal economy generally. From deficiency of exercise the function of the liver is lessened, and congestion is very liable to occur. Constipation, and its effects, as a result of this mode of life, is nearly always present. The sitting position maintained by persons of the habits under consideration, determines the blood to the hemorrhoidal vessels. From these circumstances it is very common to meet with hemorrhoidal diseases among clergymen, barristers, lawyers, those confined to the counting house, and among the working classes, the nature of whose occupations compels them to sit many hours, as dressmakers, tailors, shoemakers, and others. It is very common for individuals thus circumstanced to have the hemorrhoidal discharge occurring in a regular manner, and, when moderato in quantity, having rather a beneficial effect than otherwise, and possibly saving them from some more serious malady.

"Sometimes the hemorrhoidal flux will appear as a translation of hemorrhagic discharge from some other organ; thus arresting and keeping in abeyance morbid action that has given rise to hemoptysis, hematemesis, epistaxis, &c. Bushe mentions several instances in which this occurred, and records two cases: the one of a gentleman from Ireland, who had hemoptysis, which ceased on his being attacked with hemorrhoids, and he enjoyed good health: resorting to Paris, and being annoyed by the piles, he had them removed by Baron Dupuytren; after that he returned to America, and labored under a determination of blood to the head, of this he was relieved by leeches to the anus, and by the administration of aloes and blue pill. The other case is that of a gentleman subject to epistaxis, and who suffered from a series of cerebral symptoms, consequent on its suppression. Dr. Bushe, being consulted, prescribed stimulating pediluvia and brisk purgatives. On the patient feeling a desire to defecate, he discharged about a pint of blood per anum, to the immediate relief of the head symptoms; a regular hemorrhoidal flux continuing, he had no return of the epistaxis, or of any of the unpleasant circumstances attending its suppression.

"Mental emotions and passions, both exciting and depressing, are causes of hemorrhoids: thus anger, fear, sorrow, ennui, &c., excite a remarkable and vital action of the ganglionic nerves of the abdomen, manifested by a sense of sinking, weight, constriction and pain at the epigastrium. The result of this impression is extended to the surface of the body; the cutaneous vessels contract, inducing pallor, and the blood, driven from the surface, accumulates in the internal organs, producing various functional disorders of the stomach, derangement of the liver, jaundice, darrhœa, or hemorrhagic discharge from the rectum.

"Internal irritation from a variety of sources will produce these affections. Ascarides, which infest the lower portion of the alimentary canal, are not an infrequent cause; irritation arising from diarrhœa and dysentery will excite the hemorrhoidal discharge, and we shall observe it unfrequently as a crisis in other diseases: thus it occurs in fevers, particularly bilious and gastric fevers; also when inflammation has attacked the brain or any of the organs longed in the thoracic and abdominal cavities; and in other conditions of the system, as hypochondriasis, &c.

"Diseases of contiguous organs, by inducing an afflux of blood to the pelvic viscera, and by extension of inflammation and irritation, are common causes of hemorrhoids; we observe them accompanying disease of the prostrate gland; occurring as a consequence of stone in the bladder; the effect of stricture of the urethra, consequent on the vascular turgescence and violent straining in micturition, attendant on the aggravated forms of the latter affection.

"Excessive venery and masturbation, by producing relaxation of the system, and by determining the blood to the organs in the pelvis, produce hemorrhoidal disease.

"Certain purgatives and drastic medicines, as aloes, scammony, gamboge, black hellebore, rhubarb, the neutral salts, &c., particularly if prescribed in too frequent and too large doses, induce hemorrhoids: they act directly by irritating the mucous membrane of the rectum, and by inordinately exciting that portion of the intestine, and the lower part of the colon. Of all medicines, calomel and the other preparations of mercury have have been productive of most mischief in the affections we are now considering, as well as inducing other diseases of the digestive organs. It is not from the use of the mineral, but its general abuse, that the evil arises: the practice is justly reprobated by Drs. Copland, Elliotson, and other writers on the practice of medicine. It may, however, be questioned whether all the medicines first mentioned, when properly administered, exert much influence inducing the disease, and whether it is not rather to the state of the constitution rendering these medicines necessary that we should ascribe the local affections. They will severally readily reproduce the hemorrhoidal flux when once it has taken place, but it is not to iaferred from this that they will cause the disease, as morbid action having once occurred in a part is much more easily re-established even by slighter causes; therefore, before attributing the malady to medicines, it is essential to ascertain if there may not be other causes to which it may owe its origin."

"The most important distinction we have to consider, both in the prognosis and with regard to treatment, is the source of hemorrhage, which may be intestinal, and not the result of piles. But here a little consideration will prevent error: intestinal hemorrhage is generally a result of acute and dangerous visceral disease, and the constitutional disturbance attending it will be severe, and of marked character; it more frequently accompanies the advanced stages of malignant fevers and general cachexia. The state of the blood discharged will enable us to form a tolerably correct opinion whether it be from piles or not; when it occurs from any portion of the intertinal canal above that which is the seat of hemorrhoids it will be clotted, very dark, and mixed with the fæces and excretions, and will be passed at stool without any of the distress attending piles; nor shall we be able to detect by digital examination per anum any form of tumors or varicose state of vessels. But, on the contrary, if the hemorrhage be from piles, the blood will either precede or follow defecation, will florid in color, and fluid, with all the characters of being recently extravasated. There will also be the local symptoms attending these affections, as weight and fulness in the rectum, pain, and others which have been previously mentioned: these will be aggravated at stool; besides, examination will reveal the presence of one or more tumors, or other lesions.

"Before commencing the treatment, it is most important that a careful and minute examination of the rectum and anus should be made when a patient complains of any of the symptoms of hemorrhoidal disease: firstly, that we may arrive at a correct knowledge of the peculiar kind of tumor, and the condition of the parts, also or not of any complication; and, secondly, because the accounts given by patients themselves are frequently inaccurate, and they are too apt to dwell on any one or more of the symptoms that may be most distressing to them.

"In making an examination in the male, the patient should be directed either to lean over the back of a chair, or to lie upon a sofa on his side, with the nates projecting over the edge, and the knees drawn up; the latter position of preferable, and should always be adopted with female patients. The parts when inflamed being acutely painful, all possible gentleness must be observed, particularly if fissure of the anus exist as a complication, as slight irritation will often induce excrutiating agony. Previous to making a digital examination of the interior of the bowel, the cavity of the nail should be filled with soap, which will prevent its scratching the intestine, and the finger must be dipped in oil to facilitate its introduction; lard and unguents do not answer so well as they interfere slightly with the delicacy of the sense of the touch.

"Having become acquainted with the abnormal condition of the parts, the next consideration is, whether the hemorrhoidal affections are of a constitutional or accidental origin: it is on arriving at a just conclusion on this point that the principles of treatment must be based, and on it our success must depend. When piles have existed for a long period, have continued from youth, or the commencement of puberty, when they supervene upon or replace some serious organic or habitual affection, if they are preceded by constitutional disturbance, and succeeded by an improvement in the state of the health, if well marked indications of plethora exist, which is relieved by the accession of the hemorrhoidal flux, and if indications of congestion, or disease in any of the organs accompany or follow its suppression or interruptiou, or an hereditary predisposition exists, a constitutional nature may be inferred; and local treatment must be a secondary consideration, and not adopted till the constitutional cause has been removed or palliated : this is especially necessary if there is a predisposition, hereditary or otherwise, to apoplexy, gout, phthisis, hemoptysis, expistaxis, or other kinds of hemorrhage.

"Various authors mention instances in which a neglect of the consideration ot the constitutional origin, and the adoption of a local treatment of piles, has been followed by serious or fatal consequences Dr. Copland mentions three cases having come under his observation, in one of which fever was induced, in another apoplexy, and in a third melancholia, by the improper arrest of hemorrhoidal discharge. Mr. Howship states the case of a gentleman subject to gout, who, in opposition to proper medical advice, was induced by a charlatan to have recourse to a strong vitriolic lotion, withe the effect of arresting the hemorrhagic discharge, but the patient soon after died of gout in the stomach.

"The general treatment of hemorrhoidal affections must consist in enforcing a strict observance of moderation in diet, due attention being paid both to the quality and nature of the aliment, as well as quantity ; all stimulating food and beverarges must be forbidden, and only that allowed which is unirritating and easy of digestion: this is a matter so important, not only in the diseases herein treated of, but in all others, that it would be well to give a patient written instructions on this point, in the same manner as when medicines are directed to be taken. The bowels must be regulated, and constipation combatted, by deobstruent laxatives and stomachic aperients. If fæcal accumulation in the colon exist, these must be removed by emollient enemata: in many cases the use of O'Beirne's tube will be highly serviceable in dislodging the excrementitious matter. When the secretions and excretions of the chylopoietic viscera are depraved or deficient, means must be adopted to restore them to a healthy state; for this purpose a few grains of the blue pill with one of powdered ipecacuanha should be directed to be taken at bed time, or mercury with chalk and extract of taraxacum may be substituted; and in the morning one of the following drafts should be taken:

℞ Infusi Senuæ comp., ʒ vj;

Infusi Gentianæ comp., ʒ v;

Tincturæ Cardamomi comp., ʒ j.

Fiat haustus.

℞ Decocti Cinchonæ, Infusi Senna comp., āā ʒ jv,
Fiat haustus.

"If these are not sufficiently active, sulphate of magnesia, potassio-tartrate of soda, or sulphate of potash may be added; castor oil is a most useful laxative in these disease: a tea-spoonful of the following electuary, taken either at bed time or early in the morning, answers very well in moving the bowels once or twice:

℞ Confectionis Sennæ, Sulphuris Loti, āā ℥ j;
Pulveris Jalapæ, ʒ j;
Pulveris Zingiberis, ʒ ss;
Sodæ Potassio-tartratis, ʒ iv;
Syrupi Zingiberis, q. s.
Ut. fiat electuarium."

"When the tumors are inflamed, local depletion will generally be necessary; for the reason just urged, cupping will be more advisable than the application of leeches. If the piles are internal, and are prolapsed, they must be returned within the sphincter by gentle pressure, made by a fold of lint smeared with olive oil or spermaceti ointment; this must not be neglected, or, from vascular engorgement or constriction by the surrounding muscular fibres, mortification will probably result, occasioning severe constitutional disturbance and much suffering. Several instances of the disease being thus removed, have come under my observation. In this manner the celebrated Horne Tooke was cured of a disease he had long suffered from. Sir Benjamin Brodie, in his lectures, narrates the circumstance: "Many years ago I was dining with Dr. Pearson, and after dinner he gave an account of Horne Tooke's illness. He said that he had long labored under piles; that at last mortification had taken place; that there was no chance of his recovery; and he added, that he had that morning seen him for the last time. I remember that in the middle of this history there came a knock at the door, on which Dr. Pearson said, "Here is a messenger with an account of my poor friend's death.' However, it was some message; but by-and-by a messenger did arrive, saying that Horne Tooke was much the same, or a little better. It turned out, as I have been informed, that the piles sloughed off, and from this time he never had any bad symptom. In fact, he was, if I have been rightly informed, cured of a disease which had been the misery of his life for many years preceding, and he lived for some years afterwards."

"After the tumors have been replaced, hot poppy-head fomentations should be applied, to be succeeded by hot linseed-meal poultices. Some surgeons have advised punctures and scarifications of the inflamed and protruded piles; it is a practice that should not be adopted, being founded on erroneous principles, and will only cause the patient much annoyance, without affording the desired relief. Mr. Calvert says he saw a case of fatal hemorrhage follow the practice. Montegre and Bushe alike condemn the proceeding. After the inflammation has somewhat abated, cooling and anodyne lotions will afford great relief; an aqueous solution of opium with acetate of lead and elder flower water or rose water will answer the purpose. Enemata of cold water are beneficial in the latter stage of inflammation; the instrument used should be provided with an elastic gum jet, as one of ivory or metal will be likely to injure the tender parts. The bowels must be kept gently open by means of an aperient electuary, castor oil or other laxative."

"So long as hemorrhage appears beneficial in relieving any organ threatened with disease, it must not be arrested, but any error in the constitution or habits of the patient that tends to maintain or increase it should be corrected. When the loss of blood is frequent or large in quantity, and the patient thereby rendered weak and pale, and the irritability of the system increased, measures must be taken to moderate the flow, or to stop it entirely. In the first place, the bowels must be regulated so as to act gently every day; for this purpose, the lenitive electuary with sulphur, or sulphate of magnesia, and dilute snlphuric acid in a bitter infusion, or in an infusion of roses, may be taken early in the morning, and a teaspoonful of the confection of black pepper or Ward's paste, should be taken two or three times a day. The injection into the rectum, morning and evening, of four or six ounces of cold water, will be highly beneficial from its sedative and astringent effects. If the patient leads a sedentary life, he must take exercise daily in the open air, by which the secretions will be increased, and the circulation equalized. The food must be moderate in quantity, unstimulating in quality and taken at regular and stated intervals."

"Tumors occurring at the verge of the anus, forming external hemorrhoids, require different treatment from those which are internal to the sphincter. In the acute stage of external piles, when they are small, hot fomentations, poultices, and the medical treatment already advised, will generally succeed in relieving the symptoms; but if they be large and tense, much time and pain will be saved to the patient by making a free incision through them and evacuating the contained blood. The incision should be made with a small curved bistoury in the direction from the circumference towards the centre of the anus; immediate relief will follow, and the very slight bleeding that takes place, which is rather benefioial than otherwise, is never sufficient to cause either the patient or the sugeon any anxiety; the wound will heal by granulation, the skin contracts, and the parts are restored to their normal condition in a few days. But if this proceeding be neglected, permanent tumors will be formed in the manner previously described.

"When these exist, they should be excised, and it is the only advisable plan of treatment; if the error be committed applying ligatures to these as to internal piles, intense suffering will result, a striking example of which I witnessed in a case sometimes since. Care should be taken not to remove more of the integument than covers the tumor, or upon cicatrization of the wounds, contraction of the anus will ensue, the serious consequences of which have been referred to in Chapter IV. The usual mode of excision is by means of a pair of curved scissors: the pile, being seized with a vulsellum or pair of forceps, is to be cut off with the scissors, the incisions radiating from the circumference towards the centre of the anus. A less painful mode of removing these tumors is by a probe-pointed straight bistoury: when the tumors are large and much indurated, they slip before the edge of the scissors, rendering a second or third cut necessary; besides, a certain amount of bruising of the tissues occurs in this manner of operating, and occasions great pain unless the patient is under the influence of chloroform. In using the knife, the incisions can be made with a greater degree of exactness: each tumor is to be held with the forceps and incised at its base, the lower half of the incision being made first, that the blood may not interfere with our view. If the hemorrhoid be small it can be cut off with one stroke of the knife, but if large the preceding plan is better, as the removal of more of the integument than is necessary can be thus avoided. Should fissure of the anus coexist, it will generally heal after the excision of the tumors: slightly stimulating lotions and ointments will sometimes be advisable till the cure is complete.

"In the majority of cases it will not be necessary to interfere surgically with internal piles, if the treatment already described be steadily pursued, and the patient strictly attends to the injunctions of his medical adviser with respect to diet and exercise- Even when the tumors are large, and have existed for some time, the use of soap and water externally, night and morning, the injection of cold water or lime water after each dejection, and keeping the bowels easy, will enable the subjects of them to pass their lives in tolerable comfort. But when, notwithstanding the adoption of these means, the tumors continue affected with pain, wearing out the strength of the patient, or bleeding occurs to such an extent as to affect the constitution, producing the various symptoms that have been described, or that the tumors are constantly protruded and a profuse mucous discharge kept up, it will be advisable to remove them by surgical operation. I may be permitted to repeat that it is only when the constitution suffers from the local disease we are to remove it: and we must be careful not to do so when that disease appears beneficial in warding off those of the more important organs of the chest, head, and abdomen, which, if aggravated, might terminate fatally."

"From what has been stated, it is quite evident that excision of internal hemorrhoids is neither safe nor advisable, and that other means must be had recourse to. When the tumors are large, no plan for their removal is so effectual as the ligature, which, if properly applied, occasions but little pain, and the operation does not occupy more than a few minutes. From extensive practical experience, I can amply testify that this method is entirely free from the evil consequences mentioned by some writers, provided the necessary precautions previously pointed out have been attended to. In this belief I am supported by the evidence of gentlemen whose eminent position in the profession has afforded them a wide field for observation and practice, and whose opinions command the highest respect. In a recent consultation with Sir Benjamin Brodie, respecting a patient who was suffering from piles, complicated with prolapsus, he remarked, "The ligature is a perfectly safe proceeding." He added he had lost three patients after the operation; but two of them had albuminuria, and occurred before he had become acquainted with the pathology and important alterations in structure of the kidneys inducing that state of the urine, which the valuable researches of Dr. Bright and subsequent investigators have, since then, so ably and clearly demonstrated. In the third case, Sir Benjamin at first refused to interfere, on account of the patient's broken-down constitution, and it was only at his most urgent request, and after all the unfavorable circumstances had been pointed out to him, that he consented to perform the operation. That other fatal results have ensued upon the application of the ligature is admitted; but in these cases it will also be found the general health of the patient, or the presence of serious disease of the kidneys or other important organs, rendered the operation unadvisable. It is such cases that are adduced as militating against the practice of applying the ligature, by those who put forth some peculiar but generally not very original plan of treatment."

We have given the above copious extracts as well on account of their general correctness, as to enable the reader to judge of the value of the work.

There are, indeed, a number of points in which our own practice does not conform to the views herein expressed. For example, we regard the application of cups as positively injurious in inflamed piles. When discharges have been suppressed, or have suddenly ceased, and dangerous symptoms have supervened, the application of cups near the verge of the anus is the very best method of reproducing the discharge.

Among the most frequent causes of this affection, we have noticed the abuse of aloetics, and the use of bran in bread; and we regard the use of fruits and oleaginous substances as olive oil or bean flour, as the best articles of diet for the relief of habitual costiveness. It seems doubtful if the application of nitric acid or any similar escharotic is often admissable.

---

☞ We have received from the publishers, Lindsay & Blakestone's Physicians' Visiting List, Diary, and Book of Engagements, and also the Physician's Hand Book of Practice, by Wm. Elmer, M. D.; published by W. A. Townsend & Co.; both for the year 1861. To those practicing physicians who have once made use of this little pocket memorandum, nothing need be said of its convenience, for to such it has made itself indispensable. They are neatly bound, with tucks, and have smooth covers, which gives a good surface upon which to write, an advantage that has sometimes been sacrificed to ornament. The visiting List is for sale by S. C. Griggs & Co., at from 75 cents to $1 per copy, according to size and quality. Dr. Elmer's book is for sale by Wm. B.

Keen, for $1.25 per copy. It contains a list of diseases and their symptoms, a list of incompatibles, extemporaneous prescriptions, poisons and their antidotes, &c., &c.

Physicians from a distance, by remitting the amount indicated to either of the above houses, will receive a copy by mail, postage free.

☞ The interesting article in the present number of the *Journal*, on Spina Bifida, is from the pen of John Low, M. D., of McGregor, Iowa, the name of the author was not appended to the manuscript, and the form was in print before the omission was supplied from the letter accompanying it.

us dream of sending either of them to professional Coventry. Dr. Holmes is eminently a writer of the "sensation" type. He dearly loves a sharp antithesis, a startling paradox, a striking simile, and sentences which conclude with a proposition which crackles and snaps, or explodes like a Sharp's rifle. He is a kind of Æsculapian Beecher, who never spares friend or foe, so he may fill the pews with attentive hearers. And yet he is equally receptive, and conscious of the stirring thoughts which fill the time. Such men are eminently useful, and we are not among those who denounce active, sincere, working men, because we do not happen to fully coincide with their notions of things. On the contrary we reserve our heaviest batteries of contempt for those who rest content with "the mouldering shams of effect ages," without ever daring to put one foot in advance of the other, even in the clear sunlight of the present condition of Medical Science. The idle men, the changeless men, in these days, must fall into the rear—there is no help for it on the broadside of any parchment, whatever its origin.

We repeat we are willing to be tried by the test of the principles which Dr. Holmes advocates, albeit we protest against a single one or two conclusions which he arrives at, *in toto*. And we say this to the professor: We will wager a bottle of copaiba against an equal amount of hive syrup, that he does not, never has, and never will, practice in accordance with one proposition which he has laid down, and which medical sceptics of every kidney, from the Homœopathists up or down to "Long John," have with greedy gusto snatched up. We shall refer to, and quote it, by-and-by.

The Address commences with a glowing eulogium of deceased members of the Society. And if, as the garbled extracts and comments of outsiders would have us believe Dr. H. means to denounce the profession, the question arises why should he praise the dead for having assiduously done that which the living are still doing? Speaking of those unknown o fame, he says :

"But they have left behind them that loving remembrance which is better than fame, and if their epitaphs are chiselled briefly in stone, they are written at full length on living tablets, in a thousand homes to which they carried their ever-welcome aid and sympathy."

These are not words to be uttered of charlatans and *guessers.*

The Dr. proceeds to analyze what is called experience, upon which practice is supposed to be based, asserts that "experience must be based on the permanent facts of nature," but suggests the incontrovertible fact that "there is a changeable as well as permanent element in the art of healing," that remedies are often given, and "experience based on some prevalent belief or fashion of the time."

If this is not truth we know of no means of arriving at such a thing. We have no quarrel on hand here.

The speaker next indicates what, in his opinion, is the direction of the main intellectual current of the time, and "some of the eddies which tend to keep the Science of Medicine from moving with it, or even to carry them backwards."

"The two dominant words of our time are *law* and *average*, both pointing to the uniformity of the order of being in which we live."

"In the meantime, the great stronghold of intellectual conservatism, traditional belief, has been assailed by facts which would have been indicted as blasphemy but a few generations ago." "The more positive knowledge we gain, the more we incline to question all that has been received without absolute proof."

"The author characterizes the so-called expectant and heroic systems respectively, as Hippocratic and Themisonic, and asserts that Boston is pre-eminently "the American headquarters of the nature-trusting heresy, provided it be one." (On the contrary we assert that Boston was one of the last places to receive it, as we have occasion to know.) We must extract in full, however, a paragraph which shows conclusively that Dr. H. has "got hold of his pitcher by the right handle:"

"Nay, I will venture to say this, that if every specific were to fail utterly, if the cinchona trees all died out, and the arsenic

### BOOK NOTICE.

Dr. Holmes' book was not received in time to notice it in the proper department.

Currents and Counter-Currents in Medical Science. By Oliver Wendell Holmes, M. D., Boston; Annual Address before the Massachusetts Medical Society. 1860.

We admit having had our curiosity to see this Address very considerably excited, from the various notices it had received from the secular press. The distinction which Prof. Holmes has attained, outside of the profession, as a poet, wit, satirist, and, to some extent, as a shrewd observer of the ruling ideas of the time in various spheres of thought, prepared us for a graphic, humorous, and occasionally acute, sketch of the present state of the profession—a sort of psychologico-anatomical demonstration, by the fun-loving "Autocrat." We expected beautifully worded eulogy, spiced with keen raillery. We expected generous acknowledgment, acidified to a healthy taste by a strong infusion of wholesome truths. We did not believe that we should find, on its reading, an unmitigated jeremaid against the profession, or wholesale abandonment of its established principles, in favor of any *ism* or *pathy* whatever.

We have not been disappointed. Here and there are passages which, isolated from their surrounding, may be effectually quoted by homœopathists and *ismatics* of every and any hue, but taken as a whole, Dr. Holmes, instead of being in the minority, as he seems to think, is with the two-thirds.

We admit that one or two sentences, we in common with the vast majority or scientific physicians, wholly dissent from, but we can cull sentences equally offensive from the writings of scores of medical authors of vastly higher professional standing than the Massachusetts Professor, and yet none of mines were exhausted, and the sulphur regions were burned up, if every drug from the vegetable, animal and mineral kingdom were to disappear from the market, a body of enlightened men, organized as a distinct profession, would be required just as much as now, and respected and trusted as now, whose province should be to guard against the causes of disease, to eliminate them if possible when still present, to order all the conditions of the patient so as to favor the efforts of the system to right itself, and to give those predictions of the course of disease which only experience can warrant, and which, in so many cases, relieve the exaggerated fears of sufferers and their friends, or warn them in season of impending danger. Great as the loss would be if certain active remedies could no longer be obtained, it would leave the medical profession the most essential part of its duties, and all, and more than all, its present share of honors; for it would be the death blow to charlatanism, which depends for its success almost entirely on drugs, or at least on a nomenclature that suggests them."

The Art of Medicine, as years ago insisted upon by the writer of the present article, consists but in small part in the the administration of drugs, and the skilled physician will never think that he has done his whole duty when he has written his latin prescription.

Several "inveterate logical errors" are stated, viz:

The mode of inference *per enumerationem simplicem*, in scholastic phrase; that is, counting only favorable cases:

The *post hoc ergo propter hoc* error.

The false induction from genuine facts of observation, leading to the construction of theories which are then deductively applied in the face of the results of direct observation.

And lastly, the "assuming a falsehood as a fast, and giving reasons for it."

"Another portion of the blame rests with the public itself, which insists on being poisoned."

The fanciful and absurd prescriptions of olden time are shown to have their analogues in the present day, especially in the details of homœopathic confectionery.

Then follow several pages in which the witty Professor merely airs his humors and rhetoric, and eventually returns to sober statement of propositions again, wherein we shall attempt to follow him, and sift out if possible, the real "intent and purpose."

The author reprehends the vague and loose manner in which certain terms are employed, and defines anew as follows:

*Nature*, in medical language, as opposed to Art, means trust in the reactions of the living system against ordinary normal impressions.

*Art*, in the same language, as opposed to Nature, means an intentional resort to extraordinary abnormal impressions for the relief of disease.

The reaction of the living system is the essence of both. Food is nothing, if there is no digestive act to respond to it. We cannot raise a blister on a dead man, or hope that carminative forced between his lips will produce its ordinary happy effect.

*Disease*, dis-ease—disturbed quiet, uncomfortableness, means imperfect or abnormal reaction of the living system, and its more or less permanent results.

*Food*, in its largest sense, is whatever helps to build up the normal structures, or to maintain their natural actions.

*Medicine*, in distinction from food, is every unnatural or noxious agent applied for the relief of disease.

*Physic* means properly the *Natural* art, and Physician is only the Greek synonym of *Naturalist*.

Disease, always an effect, is always in exact proportion to the sum of its causes, and "the one prevalent failing of the Medical Art, is to neglect the causes and quarrel with the effect." In other words, ontology is general in the real doctrines which control practice. Medical men seek to expel diseases by special methods, as though each disease was some evil demon taking possession of the man, as did the devils of olden time.

The author does not believe in the degeneration theory, but believes the descendants may and often do rise to a higher standard of physical well-being than the ancestor.

Then he enunciates the startling apparent paradox:

"Many affections which art has to strive against might be easily shown to be vital to the well-being of society."

"Again, invalidism is the normal state of many organizations."

With his explanation and illustration of his meaning, there seems no objection to the proposition. But the next proposition is of very different character, and unexplained, is capable of exceedingly mischievous application:

"The *presumption* always is that every noxious agent, including medicines proper, which hurts a well man, hurts a sick one."

And germain to the same idea:

"A medicine—that is a noxious agent, like a blister, a seton, an emetic, or a cathartic—should always be presumed to be hurtful. It always is *directly* hurtful; it may sometimes be indirectly beneficial."

In nearly immediate connection follows the sentence which has excited the most professional reprehension, and awakened the highest jubilation of medical quacks and knaves, of all and sundry denominations:

"Throw out opium, which the Creator himself seems to prescribe, for we often see the scarlet poppy growing in the cornfields, as if it were foreseen that wherever there is hunger to be fed there must also be pain to be soothed; throw out a few specifics which our art did not discover, and is hardly needly to apply; throw out wine, which is a food, and the vapors which produce the miracle of anæsthesia, and I firmly believe that if the whole materia medica, *as now used*, could be sunk to the bottom of the sea, it would be the better for mankind—and all the worse for the fishes."

After illustrating his meaning upon this point, Dr. H. draws a contrast between the French and American method of practice, giving preference to the former. We need not delay to remark that even upon his own reasoning the fancied contrast is absurd, and the differences do not depend at all upon the ideas which he defends.

The remainder of the article, so far as medicine is concerned, is purely rhetorical and not argumentative. It is not a little note-worthy that, throughout the whole address, are scattered frequent indignant disclaimers, keen sarcasm, and stringent argument, against the popular folly of the time,

Homœopathy, which the disciples of that manifold imposture are very careful not to quote when they interlard their pages or conversation with choicely garbled tit-bits from the "Autocrat's famous Address." The truth is, the principles which he advocates would wipe out *that system* from existence.

The lesson which he author labors most earnestly to inculcate, is brief and simple. Rely more upon hygiene and scientific regimen, than upon drugs. Analyze experience carefully, and not be misled by fashion, fancy, or bad logic. Never use a medicine without a solidly defensible reason. The medicine which does no good, of necessity does harm. Most of the so-called medicines are unworthy of trust.

Upon the question of how many are in this latter category Dr. H. goes to an extreme which we do not care to follow, but of the essential truth of the proposition, who entertains a doubt?

In treating the sick, the administration of drugs is, we repeat, but a small proportion, and, unfortunately, the earliest part of the physician's duty.

We object utterly to the Professor's assumption that whatever hurts a well man, necessarily is to be presumed hurtful to a sick man. It is totally at variance with his own logic. In sickness the *conditions* are changed, hence causes must operate differently. Will he say that, *e converso*, whatever is beneficial to a well man is beneficial to a sick man?

We dissent wholly from his view that all medicines are directly prejudicial to the body. This is a gross confusion of terms and perversion of language. The *conditions* are the point to be observed. Some medicines may act directly and others indirectly, but the range of operation of the indirectly operating agents is narrow and diminishing. The present defect is one of the *Art* not of the *Science*.

Medicine is not, in the practice of the educated medical man, a system of *guess-work*, as the newspapers charge. It is as positive and reliable as any art can be which depends upon varying conditions and sound judgment. The great mistake has been in claiming too much for it, or attempting too much.

The author of an Introductory Address in the Medical Department of the University of Michigan, in 1853, (a production which, at the time, gave offence to some of the feebler brethren of the profession) indicated, as we still believe, the true doctrine:

"Medicine is to be looked upon and studied precisely as all other arts and sciences are looked upon and studied. The truths upon which it is assumed to be based, are to be tested as all other truths are tested; and when they cannot abide the same, let them be mercilessly discarded. A little diamond is better than a rocky mountain. If the science or art shrink by the process indicated, let it be so! Better is it to be a small but living seed than a rotten trunk, though of colossal magnitude."

We are not among the number of those who believe the address of Dr. Holmes is calculated to do the profession any injury. The sources of danger to our profession are all internal. It is the idlest folly to suppose that external attack of any sort can overcome truth. This production will awaken thought in the medical body, as a rubefacient calls back consciousness and feeling to the insensible patient. The smoke which the *pathies* and the *isms*, with the newspapers generally their ready servitors, contrived to throw around it, has gradually risen from the field, and as one of the watchmen upon the æsculapian towers, we report to the denizens; none dead, none wounded, and none missing! The field is clear for further and continuous effort to place medicine as nearly upon the level of the exact sciences, and positive arts as nature herself will allow. Not even the poetical Breakfast-Table Professor has deserted and gone over to the enemy.

